# Agarose/crystalline nanocellulose (CNC) composites promote bone marrow-derived mast cell integrity, degranulation and receptor expression but inhibit production of *de novo* synthesized mediators

**DOI:** 10.3389/fbioe.2023.1160460

**Published:** 2023-04-11

**Authors:** Marianna Kulka, Ashley Wagner, Jae-Young Cho, Syed Benazir Alam, Joy Ramielle Santos, Juan Jovel, Leshern Karamchand, Marcelo Marcet-Palacios

**Affiliations:** ^1^ Nanotechnology Research Centre, National Research Council Canada, Edmonton, AB, Canada; ^2^ Department of Medical Microbiology and Immunology 6-020 Katz Group Centre, University of Alberta, Edmonton, AB, Canada; ^3^ Department of Medicine, University of Alberta, Edmonton, AB, Canada; ^4^ The Metabolomics Innovation Centre (TMIC), 7-12 Heritage Medical Research Centre, University of Alberta, Edmonton, AB, Canada; ^5^ Northern Alberta Institute of Technology, Edmonton, AB, Canada

**Keywords:** mast cell, nanocellulose, cytokines, inflammation, IgE

## Abstract

**Introduction:** Mast cells are highly granulated tissue-resident leukocytes that require a three-dimensional matrix to differentiate and mediate immune responses. However, almost all cultured mast cells rely on two-dimensional suspension or adherent cell culture systems, which do not adequately reflect the complex structure that these cells require for optimal function.

**Methods:** Crystalline nanocellulose (CNC), consisting of rod-like crystals 4–15 nm in diameter and 0.2–1 µm in length, were dispersed in an agarose matrix (12.5% w/v), and bone marrow derived mouse mast cells (BMMC) were cultured on the agarose/CNC composite. BMMC were activated with the calcium ionophore A23187 or immunoglobulin E (IgE) and antigen (Ag) to crosslink high affinity IgE receptors (FcεRI).

**Results:** BMMC cultured on a CNC/agarose matrix remained viable and metabolically active as measured by reduction of sodium 3′-[1-[(phenylamino)-carbony]-3,4-tetrazolium]-bis(4-methoxy-6-nitro) benzene-sulfonic acid hydrate (XTT), and the cells maintained their membrane integrity as analyzed by measuring the release of lactate dehydrogenase (LDH) and propidium iodide exclusion by flow cytometry. Culture on CNC/agarose matrix had no effect on BMMC degranulation in response to IgE/Ag or A23187. However, culture of BMMC on a CNC/agarose matrix inhibited A23187-and IgE/Ag-activated production of tumor necrosis factor (TNF) and other mediators such as IL-1β, IL-4, IL-6, IL-13, MCP-1/CCL2, MMP-9 and RANTES by as much as 95%. RNAseq analysis indicated that BMMC expressed a unique and balanced transcriptome when cultured on CNC/agarose.

**Discussion:** These data demonstrate that culture of BMMCs on a CNC/agarose matrix promotes cell integrity, maintains expression of surface biomarkers such as FcεRI and KIT and preserves the ability of BMMC to release pre-stored mediators in response to IgE/Ag and A23187. However, culture of BMMC on CNC/agarose matrix inhibits BMMC production of *de novo* synthesized mediators, suggesting that CNC may be altering specific phenotypic characteristics of these cells that are associated with late phase inflammatory responses.

## Introduction

Most cell culture methods rely on two-dimensional (2D) arrangements where cells are grown either in a single monolayer on a plastic (i.e., polystyrene) surface or in suspension in liquid media. In tissues, however, most cells function within a complex three-dimensional (3D) microenvironment and interact with a protein-rich extracellular matrix and nearby cells in a spatially coordinated manner. For this reason, 3D culture scaffolds have become increasingly popular, particularly polymer-based structures that enable cells to grow upon or within them. In order for such a 3D matrix to be compatible with cell culture it must maintain a stable structure but should not interfere with normal cellular processes (also referred to as “biologically inert”). In some cases, the matrix can be biocompatible such that it facilitates or promotes normal cellular functions such as differentiation, division, chemotaxis and secretion. To be biocompatible, a matrix must have appropriate osmolarity and pH. Importantly, to enable nutrient and gas exchange the materials must also have adequate porosity.

Cellulose polymers have been used to culture stem cells and progenitor cell colonies because they maintain their structure and are generally considered to be biologically inert. Some of the earliest hemopoietic stem cell cultures were performed using methylcellulose media since this allowed for removal of individual colonies using micromanipulators (Ogawa et al., 1984) and promoted a more mature phenotype (Kurosawa, 2007). In particular, tissue-resident immune cells such as mast cells were cultured in such gel-based 3D structures for decades (Nakahata et al., 1982; Denburg et al., 1983) which allowed for manipulation of their differentiation pathways and analysis of their interactions with other cell types (Bienenstock et al., 1987; Kurosawa, 2007). Despite the importance of these semi-solid gel-based culture techniques in cell culture, there has been very little advancement in semi-solid culture substrates. Furthermore, the effect of the semi-solid media on cell function and viability has been seldom examined.

Crystalline nanocellulose (CNC) hydrogels are a novel and versatile culture substrate for cell culture because they are porous and can be chemically modified with functional groups or embedded with cell-specific biomolecules (Ma et al., 2022). Nanocellulose in the crystalline form has distinct effects on cell viability and function (Mortensen et al., 2022) and can be complexed with proteins such as casein to form composites or coupled with lysine molecules to promote cell proliferation and differentiation (Pandanaboina et al., 2021; Biranje et al., 2022). Much of the recent work with nanocellulose has relied on bacterial sources since it is low cost and easy to produce (Ugrin et al., 2021), but the presence of endotoxin in such preparations, especially when culturing immune cells, remains a concern (Moriyama et al., 2022).

In this study, we used plant-based nanocellulose crystals to assemble a CNC/agarose hydrogel matrix and determined its suitability for the culture of mouse bone marrow-derived mast cells (BMMC). Mast cells are a tissue-resident leukocyte that is absent from blood and therefore exclusively differentiates and functions in a 3D tissue environment. BMMC are not a transformed cell line and as such they exhibit aberrant growth and differentiation. BMMC are derived from wild type bone marrow stem cells and therefore are expected to respond to a 3D matrix environment like their *in vivo* counterparts. In this study, we examined the behaviour of BMMC activated with immunoglobulin E (IgE) and antigen (Ag) to crosslink high affinity IgE receptors (FcεRI) that is a classic stimulus of allergic inflammation. These responses were compared to activation using a receptor-independent stimulus (calcium ionophore A23187) which circumvents the cell membrane-proximal signaling associated with inflammatory stimuli. In both cases, BMMC production of several mediators including tumor necrosis factor alpha (TNF), a potent pro-inflammatory cytokine, was measured. The effect of the CNC/agarose matrix on cell viability was measured using three independent analyses of cell health, including measurement of NADH-dependent XTT reduction, propidium iodide (PI) incorporation, and lactate dehydrogenase (LDH) release. The expression of surface biomarkers such as the high affinity IgE receptor (FcεRI) and the stem cell factor receptor KIT/CD117 was determined using flow cytometry. Changes in mast cell transcriptome when cultured on CNC/agarose was analyzed using RNA sequencing (RNAseq).

## Materials and methods

### Materials and reagents

Spray dried crystalline nanocellulose (CNC) were kindly provided by Innotech Alberta (batch no. Comp181121-H). Agarose powder was purchased from Thermo Scientific (electrophoresis grade). All other reagents were prepared or purchased as indicated.

### Fabrication and characterization of the CNC/agarose substrate

For the transmission electron microscopy (TEM) studies, 0.02 wt% aqueous CNC solution was prepared by sonication for 10 s and deposited on a carbon-coated 400-mesh copper TEM grid (Electron Microscopy Sciences; CF400-Cu), and the excess solution was blotted after 10 s. Staining for TEM was performed by depositing one droplet of 2% uranyl acetate (UA) solution, then after 120 s, blotting and drying in air. TEM investigation was carried out on a JEOL 2200FS TEM—200 kV Schottky field emission instrument equipped with an in-column omega filter. Bright field TEM images were acquired using energy filtered zero loss beams (slit width 10 eV).

For atomic force microscopy (AFM), a clean silicon wafer substrate (1 cm^2^ × 1 cm^2^) was prepared and pre-treated with poly-L-lysine: 5 μL of an aqueous poly-L-lysine solution were deposited on a clean silicon wafer substrate (1 cm^2^ × 1 cm^2^) and incubated for 30 s, after which the sample surface was rinsed with DI water. Then, this silicon substrate was spin-coated with 0.02 wt% CNC solution at 2,500 rpm, and dried in air for 1 day. Surface morphology of CNC particles was characterized using a Veeco Dimension 3100 AFM. For optimized image quality, high resolution, soft tapping mode AFM probes (MikroMasch United States, Inc.) with low spring constants of 5.0 N/m and 1 nm radii were used.

For the cryo-scanning electron microscopy (cryo-SEM) imaging, each gel sample was placed on a small copper-based rivet, and a second rivet was carefully placed above. Then, the two-rivet ensemble was frozen by plunging vertically into liquid nitrogen (LN_2_). The frozen ensemble samples were then fractured to reveal the internal structure of or without the agarose/CNC composite. The revealed fracture surface was then sublimated for 30 min at −100°C and subsequently sputter-coated with Pt in a cryo-coater (Leica ACE600). After this, the sample was transferred to a cryo-stage SEM (Zeiss NVision40) with temperature controller and observed under an accelerating voltage of 3 keV at −100°C using the in-lens EsB (energy and angle selective backscattered electron) detector.

### Generation of the BMMC

C57BL/6 mice were sacrificed between 6 and 10 weeks old and bone marrow from their femurs was obtained by aspiration in accordance with the University of Alberta Animal Care Committee approved standard protocols and procedures. Aspirates were maintained in RPMI-1640 media (Gibco, Burlington, ON, Canada) supplemented with 4 mM L-glutamine (Gibco), 50 μM BME (Sigma-Aldrich, Oakville, ON, Canada), 1 mM sodium pyruvate (Gibco), 100 U/mL penicillin/100 μg/mL streptomycin (Gibco), 0.1 mM non-essential amino acids (Gibco), 25 mM HEPES (Gibco), 10% FBS (Gibco) and 30 ng/mL mouse recombinant IL-3 (Peprotech, Rocky Hill, NJ, United States), henceforth referred to as complete-RPMI. Cells were fed every 3–5 days. After 4 weeks, cell purity was determined by measuring expression of CD117 (KIT) and FcεRI by flow cytometry. After 4 weeks, 99% of cells were double positive for KIT and FcεRI.

### Generation of the CNC/agarose matrix and cell culture

Agarose powder was added to phosphate buffered saline (PBS) without magnesium or calcium (Gibco; 2% w/v) and heated to 100°C, while CNC powder was dissolved in PBS (2%–25% w/v), heated to 37°C for 5 min and mixed 1:1 with hot 2% agarose. Immediately after heating, 50 or 500 μL of the solution was added to the wells of a 96-well or 24-well culture plate (Thermo Fisher, Waltham, United States) respectively, and left at room temperature for 30 min to set.

For CNC/agarose composites supplemented with complete-RPMI, CNC powder was dissolved in complete-RPMI (25%), the mixture was heated to 37°C for 5 min, and mixed 1:1 with a hot 2% agarose solution prepared in complete-RPMI. The hot CNC/agarose + complete-RPMI mixture was added to the wells of a 24-well culture plate and left at room temperature for 30 min to set. BMMC were grown in complete-RPMI on top of the CNC/agarose composites (Supplementary Figure S1).

### Analysis of cell metabolic activity by measuring activity of NADH

BMMC were cultured on CNC/agarose composites (or standard untreated cell culture wells as controls) in a 96-well plate, at a density of 5,000 cells/well for 24 h at 37°C and a humidified atmosphere with 5% CO_2_. After treatment, 50 μL of XTT (sodium 3′-[1-[(phenylamino)-carbony]-3,4-tetrazolium]-bis(4-methoxy-6-nitro) benzene-sulfonic acid hydrate) with electron coupling reagent (Roche, Basel, Switzerland) was added to the wells and incubated in a humidified atmosphere at 37°C with 5% CO_2_ for 24 h to allow formation of soluble formazan salt. One hundred mL was aspirated from each well, transferred to a fresh 96-well plate and absorption was measured at 450 nm with a reference wavelength of 660 nm using a Varioskan Lux microplate reader (Thermo Fisher).

### Colorimetric analysis of LDH release

BMMC were sensitized with 500 ng/mL mouse anti-DNP IgE (SPE-7, Sigma) for 16 h or cultured in fresh media without IgE (unsensitized). The BMMC were washed with fresh media and placed onto the CNC/agarose composites (or standard untreated cell culture wells as controls) at a density of 15,000 cells/well and IgE sensitized cells were stimulated with 10 ng/mL DNP-BSA (Invitrogen) and unsensitized cells were stimulated with 1 μM A23187 for 18 h in a humidified atmosphere at 37°C with 5% CO_2_. After treatment, the cells were spun at 200 rcf (× *g*) for 5 min, and 50 μL of the supernatants were collected and aliquoted into a fresh 96-well plate. For total LDH wells, 50 μL of 0.1% Triton (Fisher, Hampton, NH, United States) was added to lyse the cells. Fifty μL of the LDH assay reagent (iodonitrotetrazolium dinucleotide sodium salt (4 mM), β-nicotinamide adenine dinucleotide sodium salt (6.44 mM), lithium L-lactate (320 mM), and TrisBase [2-amino-2-(hydroxymethyl)-1,3-propanediol (pH 8.2, 0.2 M)] was added to the wells and left to incubate in the dark at room temperature for 90 min. To stop the reaction, 50 μL of acetic acid (1 M) was added to the wells, and the absorption was measured using a Varioskan Lux microplate reader (ThermoFisher) at 490 nm with a reference wavelength of 680 nm.

### Propidium iodide exclusion assay of membrane integrity

BMMC were cultured on CNC/agarose hydrogel matrix for 18 or 24 h. Cells were removed carefully and washed twice with phosphate-buffered saline (PBS)-0.5% w/v bovine serum albumin (BSA) (Sigma-Aldrich) at 300 × *g* for 5 min at room temperature followed by resuspension in PBS-0.5% BSA at a final density of 1.5 × 10^6^ cells/mL. Cells were treated with 10 μg/mL propidium iodide (Invitrogen) for 1 h at 4°C in the dark. Fluorescence data was acquired using a CytoFlex flow cytometer (Beckman Coulter, Brea, CA, United States) equipped with an Argon ion laser (488–514 nm) and bandpass filter to enable detection fluorescence emission at 578 nm. 20,000 events at a flow rate of 30 μL/min was acquired for each sample at room temperature. As mast cells are highly granular (high side scatter, SSC) and large in size (high forward scatter, FSC) when compared to other immune cell types, such as monocytes and lymphocytes, a well-defined cell population containing relatively high FSC and SSC were gated and analyzed. This also served to preclude the cell debris with low FSC and SSC values. Data was generated using FlowJo 10.6.2 software (Becton, Dickinson and Company, Ashland, OR, United States).

### Analysis of FcεRI and KIT expression

BMMC were washed with PBS containing 0.5% BSA (Sigma) re-suspended in PBS containing 0.5% BSA (1.5 × 10^6^ cells/mL), and then incubated with 6 ng/mL rat anti-mouse CD117(KIT)-PE (eBioscience, San Diego, CA, United States) and 6 ng/mL Armenian hamster anti-mouse FcεRI-APC (eBioscience) for 1 h at 4°C. After washing with 0.5% BSA in PBS twice, cells were re-suspended in 100 μL 0.5% BSA/0.05% sodium azide in PBS and transferred to a round bottom 96-well plate. Cell samples were analyzed on a CytoFlex flow cytometer (Beckman Coulter, Brea, CA, United States). Rat IgG2b K -PE (eBioscience) and Armenian hamster IgG - APC (eBioscience) were used as isotype controls. Data was generated using FlowJo 10.6.2 software (Becton, Dickinson and Company, Ashland, OR, United States).

### RNA isolation and RNAseq analysis of BMMC transcriptome

BMMC were cultured on 12.5% CNC/agarose or agarose alone (2% (w/v) in PBS)) (three technical replicates each) at a density of 1 × 10^6^ cells/mL for 6 h and recovered from the composites by gentle aspiration. Total RNA was extracted from each of the technical replicates using TriZol (Invitrogen) and RNeasy columns (Qiagen, Hilden, Germany). On-column digestion of contaminating DNA was performed with DNase I prior to elution of RNA from the columns. Total RNA integrity was subsequently assessed using an Agilent Technologies 2,100 Bioanalyzer and each RNA isolate was confirmed to have an RNA Integrity Number (RIN) value > 9.0 (Supplementary Information). Thereafter, the mRNA in each total RNA isolate was converted into cDNA libraries and enriched *via* PCR using the Illumina TruSeq RNA Sample Preparation kit v2 (San Diego, CA, United States). The cDNA libraries (3x CNC/agarose and 3x agarose alone) were subsequently sequenced using the Illumina MiSeq Reagent kit v3 (San Diego, CA, United States).

RNAseq libraries were constructed from 100 ng of total RNA, using the TrueSeq RNA library prep kit v2 (Illumina). In brief, polyadenylated mRNAs were enriched using oligo dTs conjugated to paramagnetic beads. Enriched mRNA was chemically fragmented and reverse transcribed into cDNA. cDNAs were end-repaired, A-tailed, ligated to add adapters and finally indexed through PCR. Libraries were sequenced on a MiSeq instrument using a 75-cycle protocol that included in-instrument demultiplexing of samples. Raw sequences were inspected for quality, and no trimming was required because Q scores were on average close to 30. Sequences were pseudo-aligned against the mouse transcriptome (GRCm39) using Kallisto, with 100 bootstraps and bias correction (Bray et al., 2016a; Bray et al., 2016b). Count data were then analyzed with DESeq2 (Love et al., 2014). Briefly, RNAseq counts were modeled using a negative binomial distribution, and differential expression of transcripts were evaluated with the Ward test. *p* values were corrected with the Benjamini–Hochberg method and comparisons with corrected pValues <0.05 were considered significant.

### TNF analysis by ELISA and electrochemiluminescent multiplex analysis

BMMC were seeded at a density of 1 × 10^6^ cells/mL onto the CNC/agarose composites or standard untreated cell culture plates as controls and incubated for 18 h. BMMC were then washed and sensitized with 500 ng/mL mouse anti-DNP IgE (SPE-7, Sigma) or cultured in fresh media without IgE (unsensitized control) for 18 h. BMMC were then stimulated with either 10 ng/mL DNP-BSA (Invitrogen) or 1 μM A23187 for 18 h at 37°C. Following activation, cell suspensions were centrifuged at 200 *g* × for 5 min and cell-free supernatants were collected. Supernatants were analysed *via* commercial ELISA (ThermoFisher) and Meso Scale multiplex assays (Meso Scale Discovery, Rockville, MD, United States) following manufacturer instructions, and plates were read using the Varioskan Lux (ThermoFisher) and MESO QuickPlex SQ 120 MM (Meso Scale Discovery) plate readers respectively.

### Statistical analysis

Experiments were conducted in triplicate or quadruplicate and values represent the mean of 3 biological replicates ±standard deviation of the mean. *p*-values were determined by one-way ANOVA (between groups) with appropriate post-hoc analysis or Student t-tests.

## Results

### Preparation of the CNC/agarose matrix for cell culture analysis and effect on cell viability

**FIGURE 1:**
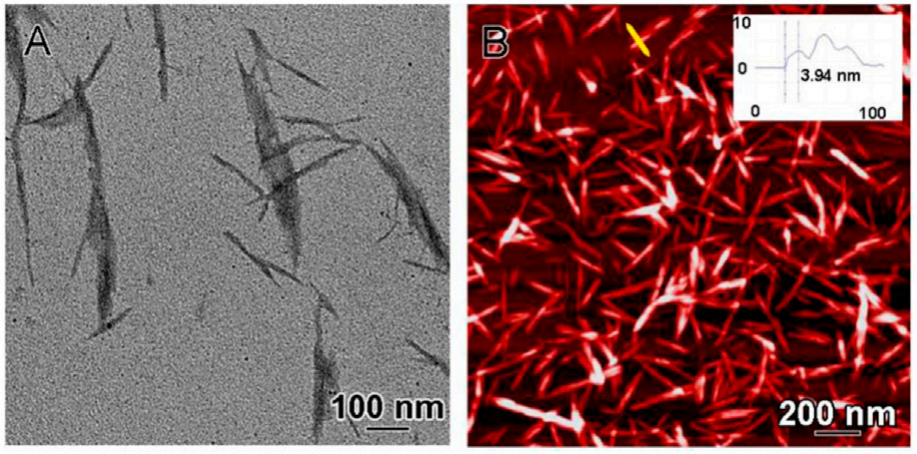
CNC morphologic and dimensional characterization. **(A)** Characterization of CNC by TEM and **(B)** AFM: inset shows the cross-sectional height profile along the yellow line which suggests multi-layered stacking of CNC—the thickness (height) of single layer is around 4 nm and the width is approximately 6 nm (inset).

The CNC used for this study was obtained as a powder and characterized using TEM and AFM (Figures 1A, B). By TEM (Figure 1A) the CNC appeared as long rod-shaped, spindle-like structures approximately 6 nm wide and 200 nm long, with an electron dense outer surface. The CNC nanorods appear as multilayered structures (see AFM data in Figure 1B), possibly through aggregation/stacking of multiple constituent CNC elements.

**FIGURE 2:**
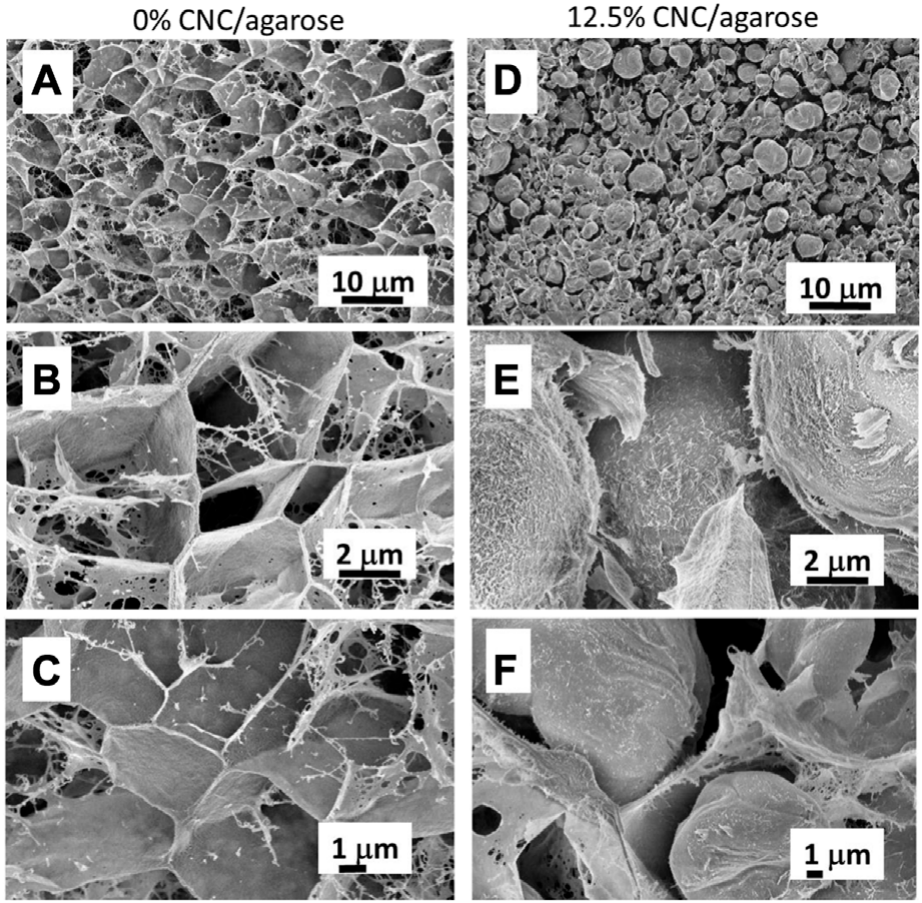
CNC/agarose and agarose gel characterization. Characterization of 1% Agarose gel without CNC (0%) **(A–C)** or with CNC (12.5%) **(D–F)** by cryo-SEM at various resolutions.

The structures of agarose and CNC/agarose gels were characterized by cryo-SEM. The agarose gel was an ordered, porous honey-comb matrix composed of polyhedral chambers with flat faces (Figures 2A–C). The characteristic dimensions of each chamber are in the 2-5 µm range, although some anisotropy is often noted along one axis. The interiors of the chambers were mainly empty, but some chambers had fibrils attached to the walls and spanning the entire chamber almost like a scaffold.

The addition of CNC significantly altered the morphology of the gel from angular structures, to spherical, ball-like structures (Figures 2D–F). The spherical structures varies in size from less than 1 µm to approximately 5 μm, and they were arranged within complex fibril-like composites (Figure 2D). Each ball-like structure has a rather smooth surface that appears increasingly fibrous with increasing magnification (Figure 2E), and these fibres are likely rich in the CNC component. The ball structures are interspersed with thin membrane-like sheets (Figure 2F).

**FIGURE 3:**
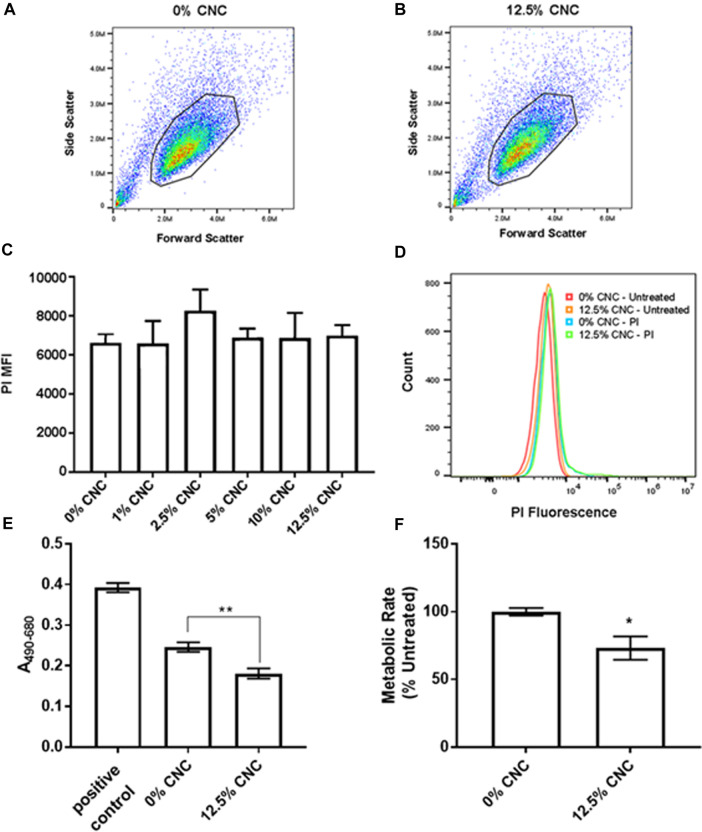
Viability and metabolic activity of cells cultured on CNC/agarose substrates. **(A, B)** Forward scatter (*X*-axis) versus side scatter (*Y*-axis) dot plot analysis of total cell population from **(A)** untreated control BMMC (0% CNC) and **(B)** BMMC cultured on 12.5% CNC/agarose substrate for 18 h depicting the gated cell population used for data analysis. **(C)** BMMCs were cultured on different CNC/agarose substrate concentrations (1%–12.5% (w/v) CNC) for 18 h, and cell membrane integrity was determined by flow cytometric analysis of propidium iodide (P1) stained cells. Graphical representation of PI mean fluorescence intensity (MFI) for BMMCs incubated on different CNC/agarose substrates relative to BMMCs that were untreated (0% CNC/agarose) and stained with PI (*n* = 4). **(D)** Histogram overlay profile of gated cells (unstained or stained with PI), from untreated control BMMCs (0% CNC/agarose) and BMMCs cultured on 12.5% CNC/agarose. **(E)** LDH release from BMMC incubated on CNC/agarose matrix for 24 h with a 0.1% triton X-100 positive control for LDH release. **(F)** BMMC were incubated on CNC/agarose matrix for 24 h and metabolic activity was determined by measuring reduction of XTT. **(E, F)** Statistical analysis used was One-Way ANOVA with Dunnett’s post-hoc test or Student T-test relative to 0% CNC, p 0.05 (*), p S 0.01 (**). Experiments were performed in triplicate and biological replicate of n-3.

The CNC/agarose matrix incorporated 12.5% (w/v) CNC. BMMC were cultured on the surface of the CNC/agarose matrix or on agarose alone for 18 h, and the size and granularity of the cell population was analyzed by flow cytometry (Figures 3A, B). Cells cultured on the agarose or CNC/agarose composites formed a normally distributed population of large and granular cells as measured by flow cytometry, similar to our previous studies (Karamchand et al., 2021; Alam et al., 2022). To determine the effect of the CNC/agarose composites on cell viability, BMMC were washed with PBS and then exposed to propidium iodide, a DNA chelating dye that fluoresces when bound to DNA (Benassi et al., 2022; Jin et al., 2022). When cells undergo necrosis or are in the later stages of apoptosis, their membrane integrities are severely compromised, and they fluoresce when exposed to propidium iodide. This effect has been routinely used as a measure of cell permeability and viability (Samarghandian et al., 2019). Flow cytometric analysis of BMMC indicated that the CNC/agarose matrix had no detectable effect on cell membrane integrity (Figures 3C, D), suggesting that the cells were healthy and their cell membranes were intact and undamaged.

Microenvironmental stress can cause metabolic changes that result in the release of intracellular enzymes such as LDH (Gabr et al., 2022). BMMC were cultured on CNC/agarose, and LDH release was measured using a standard approach as described in the materials and methods. Surprisingly, culture of BMMC on CNC/agarose resulted in a decrease in LDH release compared to cells grown in the cell culture plate alone, indicating that the CNC/agarose matrix was, in fact, promoting cell integrity (Figure 3E).

Mast cell activation can be influenced by changes in metabolic rate (Matsumura et al., 2022). To determine whether CNC/agarose composites can modify the metabolic rate of BMMC, the activity of mitochondrial NADH was assessed. When BMMC were cultured on CNC/agarose, their metabolic rate decreased by 15% (Figure 3F) compared to cells that were cultured on agarose alone.

### The CNC/agarose matrix does not change BMMC expression of FcεRI and KIT

**FIGURE 4:**
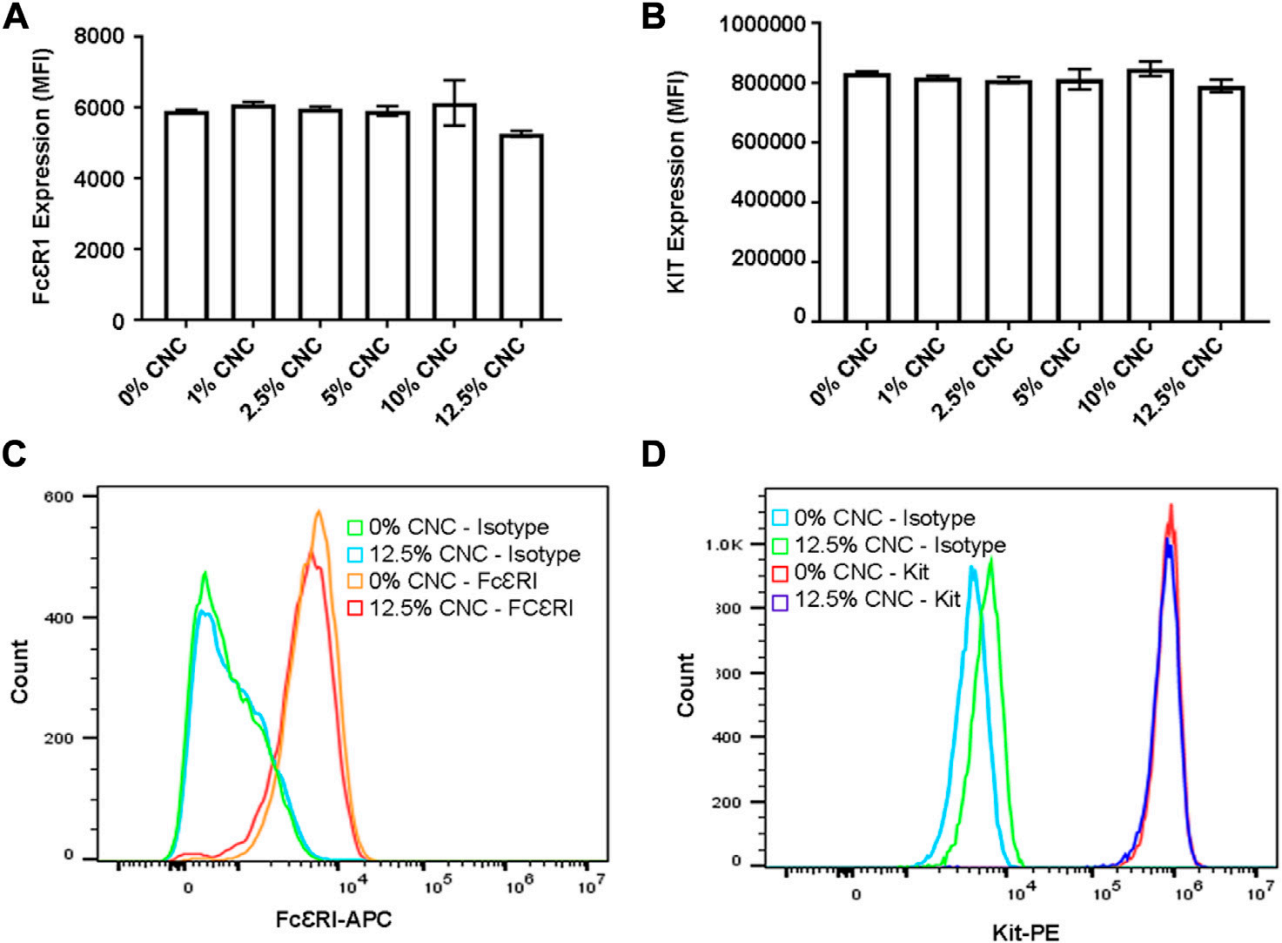
Flow cy-toinctric analysis of FceRI and Kit (0117) cell surface receptor expression post incubation on CNC/agarose substrates. BMMCs were cultured either in the absence or presence of CNC/agarose substrates (0%–12.5%) for 18 h and analyzed for FecR1 and Kit receptor expression *via* flow cytometry. Graphical representation of MFIs of untreated BMMCs (0% CNC) or BMMC incubated with 12.5% CNC/agarose substrates labeled with **(A)** anti-FccRI-APC or **(B)** anti-KIT-PE antibodies. **(C)** Histogram overlay profile of untreated BMMCs (0% CNC) or BMMC incubated with 12.5% CNC/agarose substrates and stained with anti-FccRI-APC antibody or its isotype control. **(D)** Histogram overlay profile of BMMCs incubated either alone (0% CNC) or with 12.5% CNC/agarose substrates and stained with anti-Kit-PE antibody or its isotype control. Experiments were performed with biological replicate of *n* = 4.

Two of the most important biomarkers of BMMC function and differentiation are FcεRI and KIT (CD117), the receptors for immunoglobulin E (IgE) and stem cell factor which are important for mast cell differentiation, longevity and function (Matsumura et al., 2022). When BMMC are maintained in culture, their expression of FcεRI and KIT can change over time (Matsumura et al., 2022), and changes in expression in either of these biomarkers signals undesirable changes in BMMC phenotype. When BMMC were cultured on the CNC/agarose matrix for 18 h, there were no discernable effects on the expression of FcεRI or KIT as measured by flow cytometry (Figures 4A, B) even at the highest concentration of CNC (12.5%). Although there was a slight decrease in FcεRI expression when the BMMC were cultured on the 12.5% CNC, this slight decrease was not statistically significant. A representative histogram overlay of FcεRI and KIT expression (Figures 4C, D) confirms that there were no detectable changes in either FcεRI or KIT expression, suggesting that the cells’ phenotype remains stable when they are cultured on the CNC composites.

### CNC/agarose can be supplemented to support various culture requirements

**FIGURE 5:**
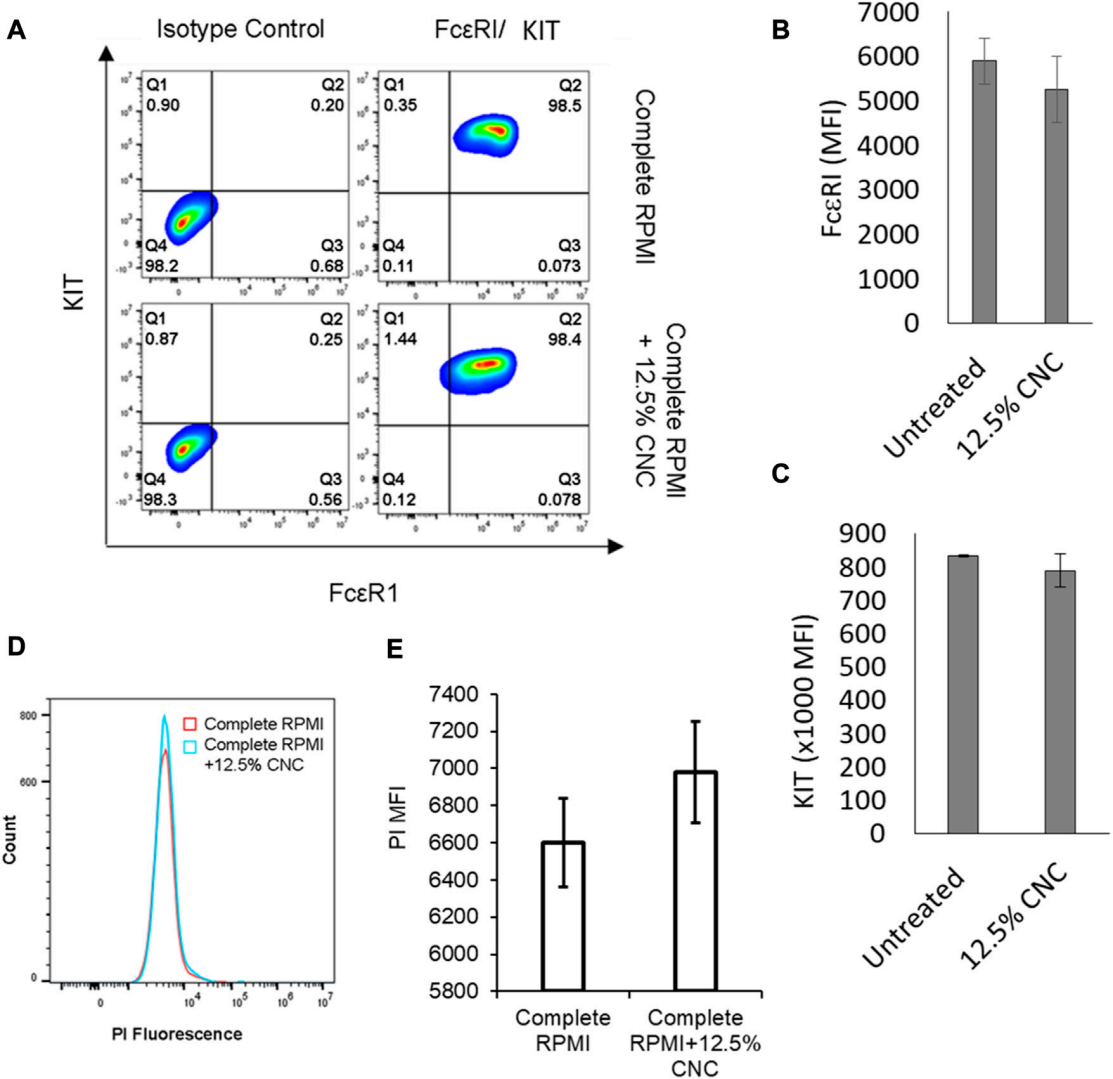
Effect of CNC/agarose substrates supplemented with complete RPMI on phenotypic expression and plasma membrane integrity of BMMC. **(A)** BMMCs were cultured in absence (Complete RPMI) or presence (Complete RPMI +12.5% CNC) of CNC/agarose substrates for 24 h. Cells were removed and analyzed for receptor expression by labeling with either the isotype controls or antibodies. KIT (*Y*-axis) versus FcsRI (*X*-axis) expression contour plots are representative of 3 independent experiments. BMMC were cultured on 0%–12.5% CNC/agarose as in **(A)** and expression of FcsRI **(B)** and KIT **(C)** mean fluorescence intensity was determined by flow cytometry (MFI; *n* = 3). **(D)** Histogram overlay profile generated from propidium iodide (PI) exclusion assay of cells treated as in **(A)**. **(E)** Graphical representation of MFI of PI-stained cells as in B; data presented is average mean fluorescence intensity (MFI) of 3 independent experiments.

To determine whether the 3D culture system modulates the expression of FcεRI and KIT and viability/cell membrane integrity compared to conventional 2D liquid culture, we constructed a CNC/agarose hydrogel prepared in complete RPMI in place of PBS. This matrix contains all the required nutrients, including supplements such as FBS and IL-3, which are required for optimal BMMC growth. BMMC grown on the CNC/agarose + complete-RPMI hydrogel resulted in relatively normal expression of FcεRI and KIT (Figure 5A) and no significant change in viability/cell membrane integrity as measured by propidium iodide staining (Figures 5B, C) when compared to BMMC cultured in liquid with complete RPMI alone.

### The CNC/agarose matrix modifies BMMC transcriptome

**FIGURE 6:**
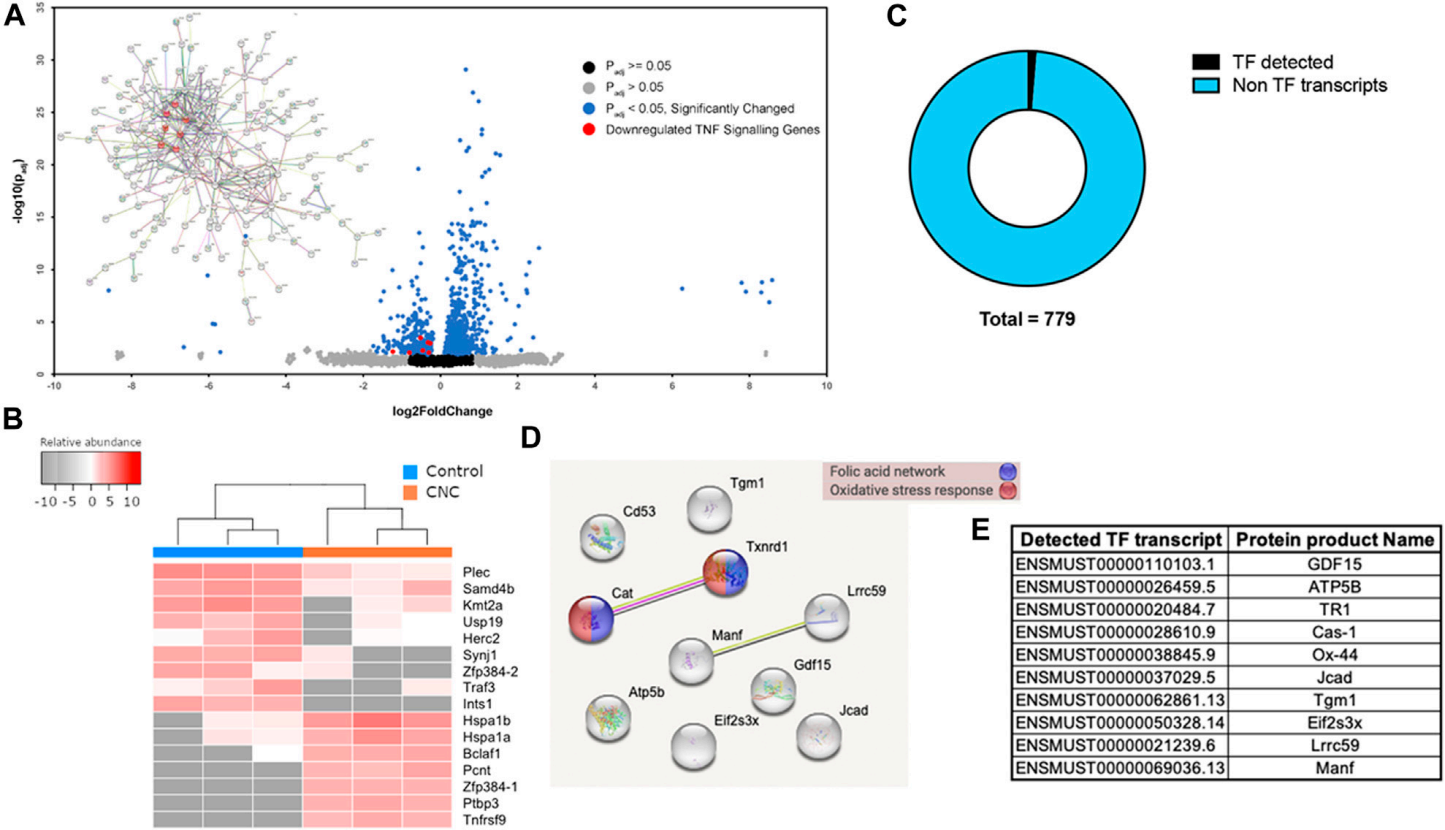
RNASeq Analysts of gene expression of BMMC grown on 12.5% CNC/agarose. **(A)** Volcano plot and (inset) STRING analysis on downregulated genes. Genes related to TNF signaling pathway highlighted in red. **(B)** Top downregulated and upregulated transcripts in CNC samples as compared to control samples. Prior to plotting, data was subjected to a regularized logarithmic transformation that also corrects for sample size. Two transcripts of gene zinc finger protein 384 (Zfp384) were found deregulated, Zfp384-1 (ENSMUST00000112427) was found upregulated with a fold change of 320, while 4384-2 (ENSMUST00000112428) was found deregulated with a fold change of 52. (1 and 2 are arbitrary suffixes added to differentiate corresponding transcripts and are not part of the HGNC ID of the gene.) **(C)** ?Fs detected in RNAseq data set. Ten Ifs were detected out of a total of 779 transcripts in the RNAseq dataset. **(D)** A STRING analysis suggests that TFs Cat (Cas-1, catalase) and Tx IMI (TRI, Thioredoxin reductase **(I)** are linked to folk acid network and oxidative stress response. **(E)** Ensembl ID for identified TF transcripts and protein names are shown.

To determine the effect of culturing the BMMC on a CNC matrix, we isolated RNA from BMMC cultured on the matrix for 6 h and examined gene expression using RNAseq (Figure 6). Principle component analysis demonstrated that the CNC/agarose replicates clustered separately from the agarose-only replicates, suggesting that culturing of BMMC on the CNC/agarose substrate significantly altered the BMMC transcriptome relative to the control (agarose only). Genes in the CNC/agarose treatment were considered to be significantly differentially expressed if they exhibited a log_2_ fold change value of ± 1 relative to the control.

This analysis revealed that more than 1000 genes were regulated by culturing the cells on CNC/agarose compared to agarose alone (Figure 6A). A volcano analysis indicates that more than 500 genes were upregulated and more than 200 genes were downregulated. TNF signalling pathway genes, such as *Birc3, Fadd, Lif, Mapk14, Ccl2* and *Fos*, were consistently downregulated as shown in red (Figure 6A). To better understand the possible associations between these genes, we analyzed functional links between them using the search tool for the retrieval of interacting genes/proteins (STRING) database. STRING analysis indicated that these genes were all interrelated, with the TNF-associated genes showing significant interrelationships (Figure 6A, red circles). In the STRING analysis, protein-coding genes *Lif, Birc3* and *Ccl2* were shown to be co-expressed while the products of genes *Fos, Mapk14, Birc3,* and *Fadd* have been experimentally determined to interact with each other.

In addition to the TNF-associated genes, several other genes were altered in the BMMC cultured on CNC/agarose and are worth noting (Figure 6B). Ten genes were downregulated or completely absent in the BMMC cultured on CNC/agarose. These genes included *Plec*, *Samd4b*, *Kmt2a*, *USP19*, *Herc2*, *Synj1*, *Zfp384-2*, *Traf3* and *Ints1*. *Synj1*, *Zfp384-2*, *Traf3* and *Ints1* which were almost completely absent in BMMC cultured on CNC/agarose. These genes were expressed by control BMMC cultured on agarose alone.

Seven genes were absent in the BMMC cultured on agarose alone, but upregulated in the BMMC cultured on CNC/agarose. These genes included heat shock proteins (*Hspa1b* and *Hspa1a*), transcription regulators (*Bclaf1*, *Pcnt*, *Zfp384-1* and *Ptbp3*) and a component of the TNF pathway (*Tnfrsf9*). Interestingly, culture of BMMC on CNC/agarose switched expression of Zfp384 transcripts from *Zfp384-2* to *Zfp384-1*.

To assess the potential contribution of transcription factors in the BMMC cytokine networks, we performed a search of transcription factors within our RNAseq transcript dataset using the *Mus musculus* database from the AnimalTFDB (Hu et al., 2019). From a total of 779 transcripts, 10 were identified as transcription factors (Figure 6C). A STRING analysis identified that 2 of these factors were involved in the folic acid network and oxidative stress response (Figure 6D). A table with the Ensembl ID and transcription factor protein names is shown in Figure 6E.

### The CNC/agarose matrix does not modify BMMC degranulation in response to IgE-dependent and IgE-independent stimuli

**FIGURE 7:**
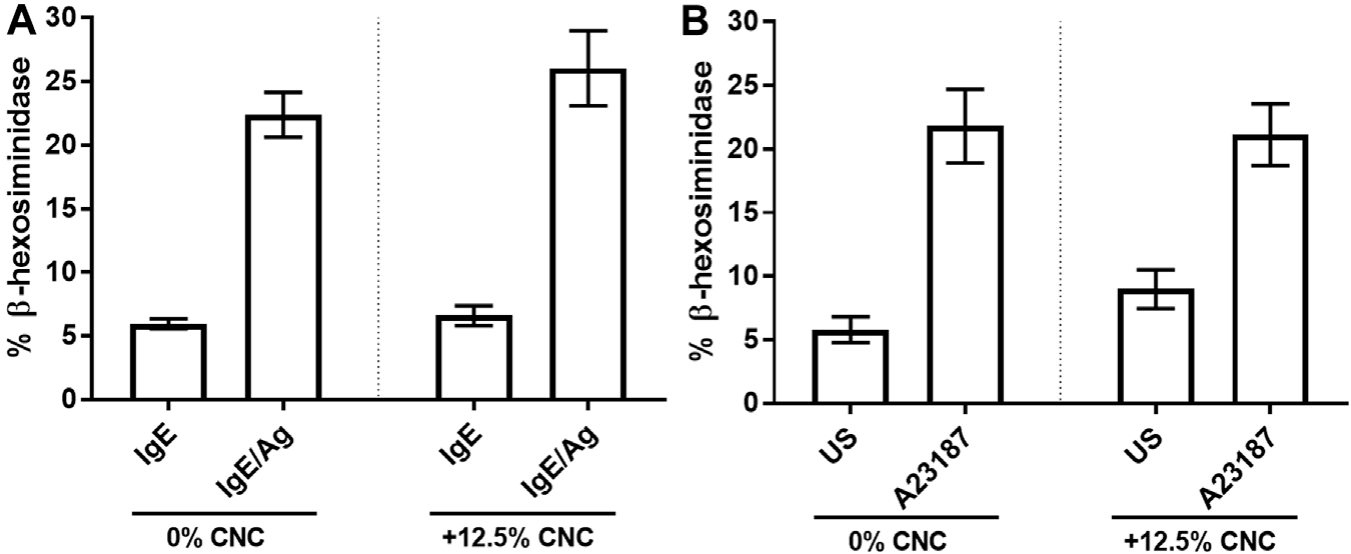
13-hexosaminidase release of BMMCs post incubation on CNC/agarose substrates. BMMC were cultured either in the absence (0% CNC) or presence (+12.5% CNC) of CNC/agarose substrates for 18 1.1, the cells were removed and activated with IgE and antigen (Ag) and 13-hexosaminidase release was measured. Cells sensitized with IgE alone (IgE) were used as a control. **(B)** BMMC were cultured as in **(A)** and activated with A23187 and 13-hexosaminidase release was measured. Uustimulated cells (US) were used as a control. Statistical analysis was performed using One-Way ANOVA with Tukey post-hoc test relative to activated cells incubated in the absence of CNC (0% CNC). Experiments were performed in quadruplicate with biological replicate of *n* = 3.

One of the most critical requirements of a suitable 3D cell culture matrix is its ability to maintain normal cell function, particularly when cells are activated with stimuli. BMMC cultured on CNC/agarose or agarose alone were activated with IgE and antigen (IgE/Ag) which crosslinks their surface FcεRI receptors and initiates a series of signaling events leading to degranulation and mediator release. Although CNC/agarose appeared to increase the degranulation response of the BMMC when compared to BMMC cultured on agarose alone, this effect was not statistically significant (Figure 7A).

Next, BMMC that had been cultured on CNC/agarose or agarose alone were stimulated with A23187, a calcium ionophore which increases intracellular calcium concentration and initiates degranulation and the release of mediators stored in granules, including the lysosomal enzyme β-hexosaminidase. Degranulation of BMMC in response to A23187 did not change when the BMMC were cultured on agarose or CNC/agarose (Figure 7B). These results suggest that degranulation and the release of stored mediators is not affected by culture on the CNC/agarose hydrogel.

The CNC/agarose matrix inhibits BMMC production of cytokines, chemokines and other mediators when activated with IgE-dependent and IgE-independent stimuli.

BMMC cultured on CNC/agarose matrix were stimulated with IgE and antigen (IgE/Ag) which crosslinks their surface FcεRI receptors and also initiates a series of signaling events leading to the gene transcription and protein production of several pro-inflammatory mediators. Electrochemiluminescent multiplex screen analysis of several mediators indic7ated that BMMC activated with IgE/Ag produced IL-6 and MCP-1/CCL2 but not IL-4, IL-13, MMP-9 or RANTES (Supplementary Figures S2A–G). IgE/Ag-induced production of IL-5 and MCP-1/CCL2 was greatly reduced in BMMC that had been cultured on CNC/agarose, with almost complete absence of these cytokines in CNC/agarose-cultured BMMC (Supplementary Figures S2C, E). Interestingly, BMMC stimulated with A23187 produced IL-1β, IL-4, IL-6, IL-13, MCP-1/CCL2, MMP-9 and RANTES. However, BMMC cultured on CNC produced notably lower amounts of all of these mediators, especially IL-4, IL-13, MMP-9 and RANTES which were almost completely absent in CNC/agarose cultured BMMC (Supplementary Figures S2B, D, F, G). This data suggests that CNC has a profound inhibitory effect on production of these mediators by BMMC.

**FIGURE 8:**
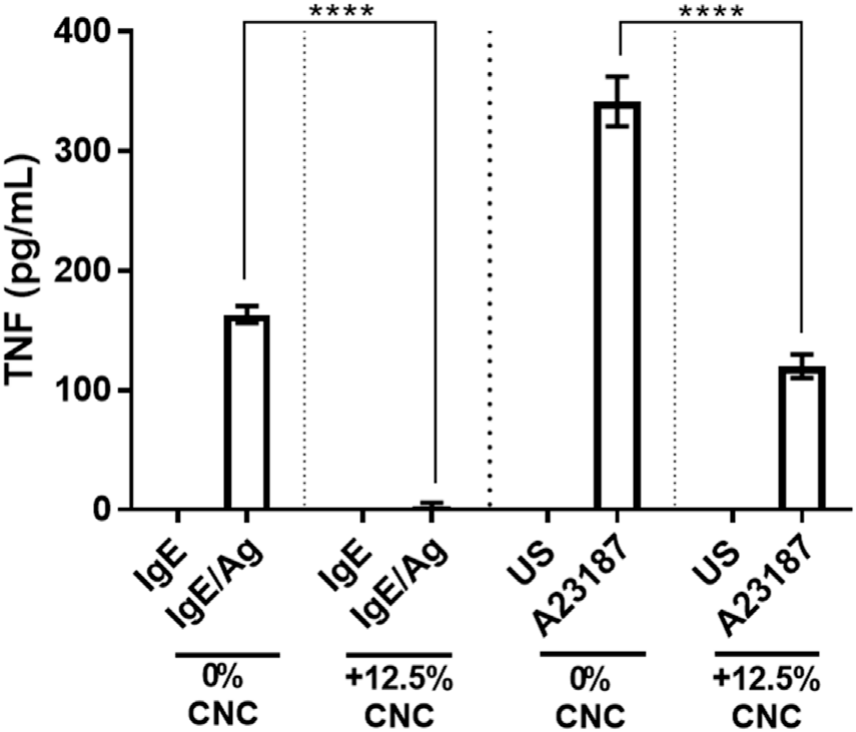
CNC/agarose matrix effect on BMMC mediator release. (A) BMMC were cultured in the absence (0)% CNC) or presence (12.5% CNC) of CNC/agarose substrates for 18 h, removed and activated with either IgE and antigen (Ag) where cells sensitized with IgE alone (IgE) were used as a control, or A23187 where unstimulated cells (US) were used as a control and their production of TNF was measured by ELISA. Statistical analysis was performed using One-Way ANOVA with Tukey post-hoc test relative to activated cells incubated in the absence of CNC (0% CNC), *p* < 0.0001 (****). Experiments were performed in triplicate with biological replicate of n-3.

BMMC produce a significant amount of TNF that was not analyzed in the electrochemiluminescent multiplex analysis. To test for TNF production, BMMC were cultured and stimulated as described above, and TNF production was analyzed using ELISA. BMMC cultured on CNC/agarose released significantly less TNF when activated with IgE/Ag compared to BMMC cultured on agarose alone and activated with IgE/Ag (Figure 8). Similarly, BMMC cultured on CNC/agarose produced less TNF when activated with A23187 compared to BMMC cultured on agarose alone (Figure 8).

## Discussion

Culture of cells on semi-solid media has been used to alter cell phenotype and allow for better manipulation of cells during culture. Although nanocellulose and its various forms have been used in many commercial products, the material’s effects on cell culture and specifically cell phenotype, genotype and function are still poorly understood (Endes et al., 2016). Our analysis compared the culture of BMMC on agarose and CNC/agarose to better understand the influence of CNC on BMMC phenotype and function. Our analysis indicated that culture of BMMC on CNC/agarose leads to retention of FcεRI and KIT/CD117 expression and maintenance of membrane integrity, but a reduction in metabolic activity, altered gene expression and reduced mediator production upon activation with A23187 and IgE/Ag. These data suggest that culture with CNC is changing BMMC function without altering their phenotype and highlights the importance of semi-solid media composition on generating physiological cellular phenotypes.

EM analysis of the CNC/agarose matrix suggests that the gel forms a porous and complex structure, and that the addition of CNC significantly alters the morphology of the gel, creating ball-like structures that possibly contain liquid. This porous structure would allow for the diffusion of nutrients and gases, perhaps providing a more amenable microenvironment for the cells. Although the cells were placed on top of this matrix, they likely experience slight changes in nutrient and waste product concentrations as they diffuse into the matrix below them. This may explain why the BMMC showed a general lack of change in viability and membrane integrity (Figure 3), suggesting that the cells were healthy overall and did not experience any adverse effects while cultured on CNC/agarose. Although there was a slight decrease in metabolic activity, this could be due to slight nutrient changes at the surface of the CNC/agarose gel. This could also be due to changes in oxygen levels leading to oxidative stress which can influence mitochondrial function. There are very few studies that have examined the effect of nanocellulose on oxidative stress, but some *in vivo* research has suggested that various forms of nanocellulose and cellulose nanocrystal-derived nano-onions can induce a slight increase in reactive oxygen species (ROS) generation in gut epithelium (DeLoid et al., 2019), and modulate oxidative stress in HEK293 cells (Abidi et al., 2021). Cationic derivatives of crystalline crystalline nanocellulose have induced mitochondrial ROS and ATP release in a mouse macrophage cell line (J774A1) and peripheral blood mononuclear cells (Sunasee et al., 2015). Both fibrillar and crystalline nanocellulose induce ROS generation (Wang et al., 2019). Nanocellulose fibrils have a more deleterious effect on oxidative stress in A549 lung epithelial cells compared to CNC, especially when CNC are incorporated into a gel (Menas et al., 2017). Wistar rats treated with nanocellulose showed an elevation in biomarkers associated with renal oxidative damage and increased expression of inducible nitric oxide synthase and COX-2, suggesting inflammation (Adewuyi et al., 2018). Yet, it remains to be determined whether CNC can directly affect NADPH generation in BMMC.

One surprising observation was that although BMMC cultured on CNC/agarose were able to degranulate normally (Figure 7), when activated with either A23187 or IgE/Ag, they produced significantly less *de novo* synthesized mediators that require transcription and translation (Figure 8). This decrease in mediator-producing capacity appeared to be reasonably universal such that the production of every mediator that was measured was significantly reduced. This is in contrast to A549 epithelial cells that increase cytokine and chemokine production when exposed to CNC—increasing their production of IL-6, IL-8, MCP-1, IL-1β, IL-12p70 and G-CSF after 72 h of exposure (Menas et al., 2017). In fact, several inflammatory mediators were produced by A549 cells, suggesting a general upregulation of several transcription pathways in these cells (Menas et al., 2017). In contrast, our RNAseq analysis indicated that there was a more balanced response to CNC by BMMC—with some genes upregulated, but others downregulated, suggesting that BMMC were choosing specific responses to their new microenvironment, rather than reacting ad-hoc.

BMMC cultured on CNC/agarose upregulated 7 genes that were effectively absent in cells that were cultured on agarose alone. These genes include heat shock proteins *Hspa1a* and *Hspa1b* which serve as chaperones for other proteins involved in cytoskeleton function and have been associated with atrial fibrillation and colorectal polyp formation (Szczuka et al., 2021; van Marion et al., 2021). These proteins appear to be essential in cellular responses to changes in microenvironment and therefore it is unsurprising that their expression is induced in BMMC cultured on CNC/agarose. *Bclaf1*, another gene that was upregulated in BMMC cultured on CNC/agarose as compared to control, is a transcription factor regulator that has a wide range of biological functions, including extracellular vesicle production and DNA damage responses (Jin et al., 2022). The role of *Bclaf1* in BMMC function and gene transcription has not been explored but it is likely that it is mediating some overall changes in cell phenotype.

BMMC cultured on CNC/agarose also upregulated several transcription regulators and regulators of cell division (*Pcnt*, *Zfp384-1* and *Ptbp3*). *Pcnt* encodes pericentrin, a protein found in centrosomes which regulate the assembly of microtubules during cell division. Pericentrin function in mast cells or the effect of CNC on its expression is unknown. However, pericentrin is an essential protein in spindle formation and cell cycle progression of cells (Chinen et al., 2021) and thus may be an important component of BMMC cell division in this new microenvironment.

Ptbp3 encodes a protein that is critical in RNA splicing and transcriptional regulation (Xie et al., 2022). The PTBP3 protein mediates TGF-β-induced epithelial-to-mesenchymal transition (Dong et al., 2022) and can serve as an immunomodulatory biomarker of cancer (Chen et al., 2022). Its expression and function in mast cells is unknown.

Interestingly, culture of BMMC switched expression of the zinc finger transcription factor *Zfp384* from the *Zfp384-2* to the *Zfp384-1* isoform, suggesting that the different isoforms have opposite functions in these two microenvironments. To our knowledge this type of switch in expression has not been described before. ZFP384 is also known as nuclear matrix protein 4 (NMP4) and was originally identified as a parathyroid hormone-responsive nuclear matrix architectural transcription factor that regulates gene activity by bending DNA (Torrungruang et al., 2002; Childress et al., 2015; Rump et al., 2016). Recent work has shown that ZFP384/NMP4 may directly bind IL-1β (Nakamoto et al., 2016) and bind to the promoter and/or non-coding sequences of chemokines CCL2, CCL7 and CXCL1 in lung epithelial cells and bone marrow-derived macrophages (Yang et al., 2021).

BMMC cultured on CNC/agarose also upregulated a component of the TNF pathway, TNF receptor superfamily member 9 (*Tnfrsf9*). TNFRSF9 is an important regulator of mast cell function in several pathologies, including Lewis lung carcinoma in mouse models (Wensman et al., 2012), and TNF production and signaling in mast cells has been recognized as an important aspect of their function for decades. To follow up on this observation, TNF production by BMMC cultured on CNC/agarose was determined. As with most of the mediators tested, BMMC cultured on CNC/agarose produced less TNF than BMMC cultured on agarose, suggesting that CNC/agarose modified the TNF pathways in complex ways—upregulating some components while downregulating others. Interestingly, this change in response did not affect degranulation, since BMMC cultured on CNC/agarose degranulated similarly to BMMC cultured on agarose alone (Figure 7).

In conclusion, our data indicate that culture of BMMC on CNC/agarose leads to maintenance of mast cell integrity, degranulation and biomarker expression but inhibits their production of *de novo* synthesized mediators. This change in phenotype appears to be specific to newly synthesized mediators that require transcription and translation, suggesting that CNC/agarose modifies mast cells such that these pathways are fundamentally altered. Although the cells produced fewer mediators, their expression of mast cell receptors, CD117 and FcεRI were unaltered, demonstrating that the differentiation of these cells remain constant. Overall, our data suggest that CNC/agarose may be a viable option for the culture of BMMC in a 3D culture system, although their production of mediators may be altered.

## Data Availability

Raw sequencing data associated to this study is publicly available at the SRA database of NCBI under the accession number PRJNA938749.
