# Supplementary material for: Agarose/crystalline nanocellulose (CNC) composites promote bone marrow-derived mast cell integrity, degranulation and receptor expression but inhibit production of de novo synthesized mediators
Source: Front Bioeng Biotechnol. 2023 Apr 11;11:1160460. doi: 10.3389/fbioe.2023.1160460 (PMC10126518; doi:10.3389/fbioe.2023.1160460)
Supplement: Supplementary file 1 [file Presentation1.pdf]

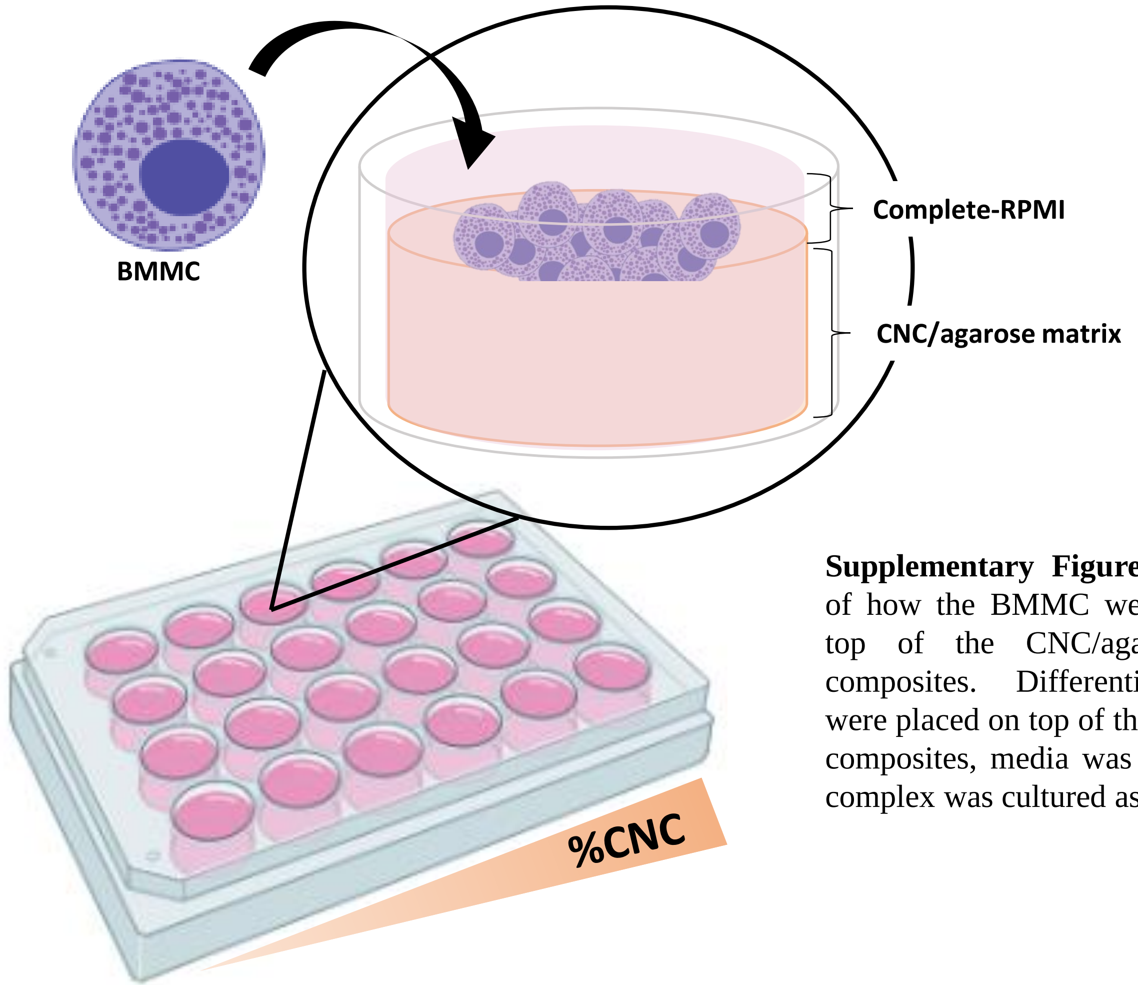

Supplementary Figure 1. Schematic of how the BMMC were cultured on top of the CNC/agarose (12.5%) composites. Differentiated BMMC were placed on top of the CNC/agarose composites, media was added and the complex was cultured as indicated.

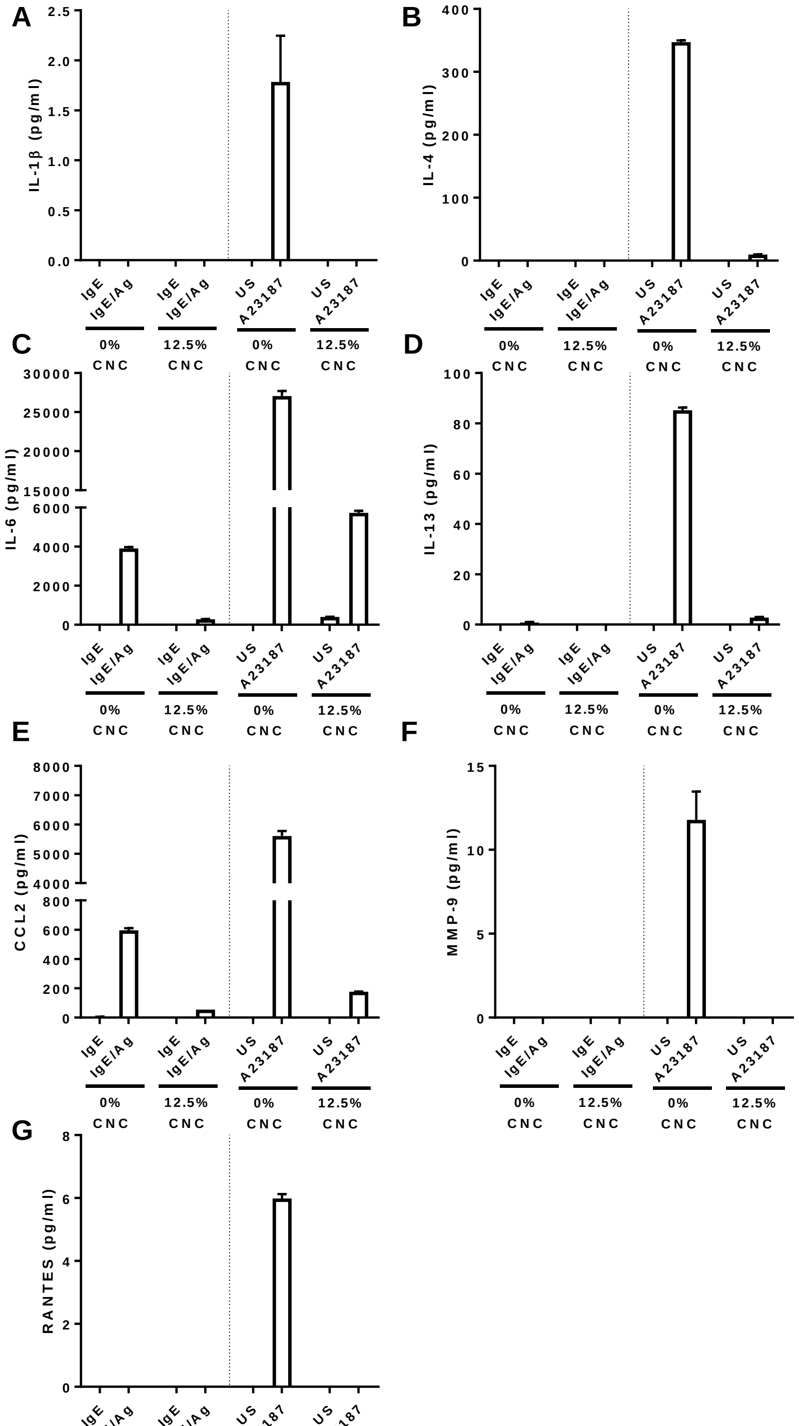

Supplementary Figure 2. Electrochemiluminescent multiplex screen analysis of BMMC post incubation on CNC/agarose substrates (12.5%). BMMC were cultured in the absence (0% CNC) or presence (12.5% CNC) agarose/CNC substrates for 18 hr removed and stimulated with either IgE and antigen (Ag) where cells sensitized with IgE alone (IgE) were used as a control, or A23187 where unstimulated cells (US) were used as a control and mediator release was measured using electrochemiluminescent multiplex analysis for (A) IL-1β, (B) IL-4, (C) IL-6, (D) IL-13, (E) CCL2, (F) MMP-9 and (G) RANTES.
